# Exploring educational inequalities in hypertension control, salt knowledge and awareness, and patient advice: insights from the WHO STEPS surveys of adults from nine Eastern European and Central Asian countries

**DOI:** 10.1017/S1368980023000356

**Published:** 2023-12

**Authors:** Katerina Maximova, Enrique Loyola Elizondo, Holly Rippin, João Breda, Francesco P Cappuccio, Morteza Hajihosseini, Kremlin Wickramasinghe, Irina Novik, Vital Pisaryk, Lela Sturua, Ainura Akmatova, Galina Obreja, Saodat Azimzoda Mustafo, Banu Ekinci, Toker Erguder, Shukhrat Shukurov, Gahraman Hagverdiyev, Diana Andreasyan, Carina Ferreira-Borges, Nino Berdzuli, Stephen Whiting, Natalia Fedkina, Ivo Rakovac

**Affiliations:** 1 MAP Centre for Urban Health Solutions, Li Ka Shing Knowledge Institute, St. Michael’s Hospital, 209 Victoria Street, Toronto, ON M5B 1T8, Canada; 2 Dalla Lana School of Public Health, University of Toronto, Toronto, Canada; 3 World Health Organization European Office for the Prevention and Control of Non-Communicable Diseases (NCD Office), Division of Country Health Programmes, WHO Regional Office for Europe, Moscow, Russia; 4 WHO Collaborating Centre for Nutrition, University of Warwick, Coventry, UK; 5 Division of Health Sciences, Warwick Medical School, University of Warwick, Coventry, UK; 6 School of Public Health, University of Alberta, Edmonton, Canada; 7 Republican Scientific and Practical Center of Medical Technologies, Informatization, Management and Economics of Public Health (RSPC MT), Minsk, Belarus; 8 National Center for Disease Control and Public Health (NCDC) of Georgia, Tbilisi, Georgia; 9 Department of Public Health, Ministry of Health, Bishkek, Kyrgyzstan; 10 Department of Social Medicine and Management, Nicolae Testemitanu State University of Medicine and Pharmacy, Chisinau, Republic of Moldova; 11 State Research Institute of Gastroenterology, Ministry of Health and Social Protection of Population, Dushanbe, Republic of Tajikistan; 12 Department of Chronic Disease and Elderly Health, General Directorate of Public Health of Ministry of Health of Turkey, Ankara, Turkey; 13 WHO Country Office in Turkey, Ankara, Turkey; 14 Central Project Implementation Bureau of the Health-3 Project, Tashkent, Uzbekistan; 15 Public Health and Reforms Center, Ministry of Health, Baku, Azerbaijan; 16 National Institute of Health, Ministry of Health, Yerevan, Armenia

**Keywords:** Hypertension control, Salt knowledge and perceptions, Salt consumption behaviours, Educational inequalities, Physician’s advice, WHO STEPS

## Abstract

**Objective:**

To inform strategies aimed at improving blood pressure (BP) control and reducing salt intake, we assessed educational inequalities in high blood pressure (HBP) awareness, treatment and control; physician’s advice on salt reduction; and salt knowledge, perceptions and consumption behaviours in Eastern Europe and Central Asia.

**Design:**

Data were collected in cross-sectional, population-based nationally representative surveys, using a multi-stage clustered sampling design. Five HBP awareness, treatment and control categories were created from measured BP and hypertension medication use. Education and other variables were self-reported. Weighted multinomial mixed-effects regression models, adjusted for confounders, were used to assess differences across education categories.

**Settings:**

Nine Eastern European and Central Asian countries (Armenia, Azerbaijan, Belarus, Georgia, Kyrgyzstan, Republic of Moldova, Tajikistan, Turkey and Uzbekistan).

**Participants:**

Nationally representative samples of 30 455 adults aged 25–65 years.

**Results:**

HBP awareness, treatment and control varied substantially by education. The coverage of physician’s advice on salt was less frequent among participants with lower education, and those with untreated HBP or unaware of their HBP. The education gradient was evident in salt knowledge and perceptions of salt intake but not in salt consumption behaviours. Improved salt knowledge and perceptions were more prevalent among participants who received physician’s advice on salt reduction.

**Conclusions:**

There is a strong education gradient in HBP awareness, treatment and control as well as salt knowledge and perceived intake. Enhancements in public and patient knowledge and awareness of HBP and its risk factors targeting socio-economically disadvantaged groups are urgently needed to alleviate the growing HBP burden in low- and middle-income countries.

The global target of a 25 % relative reduction in the prevalence of raised blood pressure (BP) by 2025 adopted by the WHO^(1)^ is supported by all European countries^(2)^. High blood pressure (HBP) or hypertension is a major modifiable risk factor for CVD and a leading risk factor for global deaths^(3)^. In the WHO European Region, 54 % of CVD burden is attributable to HBP^(4)^ and 28 % of adults have HBP (≥140/90 mmHg), with the highest prevalence in Eastern European and Central Asian countries where it frequently surpasses 40 %^(3)^. The HBP burden has doubled worldwide since 1990, with most of the increases occurring in low- and middle-income countries. This burden is projected to further increase by 60 % by 2025, disproportionately affecting low- and middle-income settings^(3)^. Despite the high prevalence, many individuals are unaware they have HBP, and among those who are aware, many are untreated or uncontrolled, particularly in low- and middle-income countries^(3,5)^.

A substantial body of evidence convincingly implicates excess salt (sodium chloride) intake in the pathogenesis of HBP and the atherosclerotic process underlying CVD^(6)^. Current levels of dietary salt consumption grossly exceed human physiological needs^(7)^, with excess salt consumption being highly prevalent worldwide^(8)^. In countries of the WHO European Region, average daily salt intake is 2–3 times above the maximum daily recommended intake of <5 g/d and is particularly high in Central Asian and Eastern European countries^(9)^. Improved recognition of the importance of salt has been identified as one of the major public health and medical challenges in the prevention and treatment of HBP and CVD.

Population-wide reduction of dietary salt consumption is considered to be the most cost-effective intervention to help achieve the global target of a 30 % relative reduction in mean population intake of salt/sodium^(10,11)^ and has been advocated by the WHO as a crucial strategy for tackling the global burden of disease^(8,12)^. Several countries have adopted formal policies aimed at reducing salt intake in the general population, with implementation of comprehensive national programmes shown to lead to approximately a 10 % reduction in salt intake and significant reductions in population BP levels, the incidence of CVD events, and CVD mortality^(13)^. In the WHO European Region, twenty-six out of fifty-three countries had an operational salt reduction policy in 2013, although countries in Eastern Europe and Central Asia are lagging behind^(14)^. While comprehensive and multifaceted efforts that include food reformulation, front-of-pack labelling, marketing limitations and surveillance are needed to lower dietary salt consumption, improvements in salt knowledge and perceptions, combined with primary health care counselling and advice to reduce salt intake, are essential components of all successful population-wide salt reduction strategies^(15)^.

Promoting health and well-being is a core priority of the WHO General and European Programme of Work^(16,17)^, and measuring and reducing socio-economic inequalities, leaving no one behind, is a major goal of the European Programme of Work 2020–2025^(17)^. Socio-economic inequalities (whether based on education, income or occupation) in non-communicable disease (NCD) morbidity and mortality have been previously documented, with CVD and HBP disproportionately affecting socio-economically disadvantaged groups^(18)^. However, little is known about socio-economic inequalities in HBP awareness, treatment and control, salt knowledge, perceptions and consumption behaviours, and physician’s advice on salt reduction among adults across different socio-economic groups^(9)^, and virtually no evidence on socio-economic gradients exist in Eastern Europe and Central Asia. In this study, we examined educational inequalities in HBP awareness, treatment and control, physician’s advice on salt reduction, and salt knowledge, perceptions of salt intake and salt consumption behaviours among adults from nine Eastern European and Central Asian countries. We ask the question whether adults with lower education will have (a) lower prevalence of HBP awareness, treatment and control, (b) lower prevalence of physician’s advice on salt reduction, and (c) poorer salt knowledge, perceptions and behaviours, compared to those with higher education.

## Methods

### Study population

Data from nine countries in Eastern Europe and Central Asia (Armenia, Azerbaijan, Belarus, Georgia, Kyrgyzstan, Republic of Moldova, Tajikistan, Turkey and Uzbekistan) were collected in cross-sectional, population-based national household surveys through the WHO STEPwise approach to Surveillance (WHO STEPS). The WHO STEPS is the WHO-recommended framework for NCD surveillance. The STEPS survey methods are described in depth in the WHO STEPS Surveillance Manual^(19)^, and details are given in the individual country reports^(20)^. Briefly, a multi-stage clustered sampling design was employed to draw a nationally representative sample in each country. Primary, secondary and tertiary sampling units represented geographical divisions (e.g. provinces, regions and villages). A limited number of households (usually 12–20) in each primary selection unit were selected, and one adult per household was randomly chosen for participation in the survey. Due to the ways data were collected in each country, the study population included adults aged 25–65 years, 15–69 years and 18–65 years in Kyrgyzstan, Turkey, Uzbekistan, respectively, and 18–69 in six other countries (Table 1). Participation was voluntary, and informed consent was obtained using country-specific language forms. In addition, to protect the confidentiality of collected and archived data, records were anonymised with unique identifiers assigned to participants. Ethical approval was obtained in each country before survey administration.

**Table 1:** Overview of WHO STEPS surveys in nine Eastern European and Central Asian countries

Country	Income level^*^	Survey year	*n* surveyed	Response rate (%)	Age group (years)	Female (%)	*n* analysed
Armenia	UMIC	2016	2376	42	18–69	69	1878
Azerbaijan	UMIC	2017	5616	97	18–69	59	4700
Belarus	UMIC	2016	5017	87	18–69	58	4224
Georgia	LMIC	2016	4212	76	18–69	70	3399
Kyrgyzstan	LMIC	2013	2640	100	25–65	64	2623
Republic of Moldova	LMIC	2013	4823	84	18–69	62	3983
Tajikistan	LMIC	2016	2718	99	18–69	60	2237
Turkey	UMIC	2017	6053	70	15–96	60	4208
Uzbekistan	LMIC	2014	3856	89	18–65	60	3203

LMIC, lower-middle-income country; UMIC, upper-middle-income country.

* The World Bank income level in the year of the survey.

### Survey instrument

Data were collected using the STEPS survey instrument, which is both standardised and adaptable to each country’s priorities, needs and resources^(21)^. The information was collected using standardised questionnaire via face-to-face interviews at the participant’s home by interviewers trained by the WHO. The questionnaire includes questions about socio-demographic characteristics (age, sex, level of education, marital status and work status), and NCD risk factors such as consumption of vegetables and fruit, salt-related behaviours and perceptions, physical activity, history of risk factor measurements and diagnosis for selected NCD, and advice from health care professionals regarding risk factors. Standard WHO and internationally recognised definitions and measures are used in order to maintain comparability. The interview is followed by basic physical measurements, including height, weight, waist circumference and BP, collected by trained interviewers at respondent’s home^(19,20)^.

### Measures

BP was measured at the end of the home questionnaire, using a standardised protocol. Trained interviewers assessed BP in the sitting position, on the left arm, after the participant had voided their bladder, fasted and refrained from drinking coffee or smoking for >30 min and following a 5-min rest after interview, using digital sphygmomanometers and universal sized cuffs. Three readings were taken, at least 3 min apart, and the second and third readings were used to calculate an individual’s mean value. Five hypertension awareness, treatment and control categories were created as follows: normotensive (BP < 140/90 mmHg, not on HBP medication); hypertensive treated, controlled (BP < 140/90 mmHg, on HBP medication); hypertensive treated, uncontrolled (BP ≥ 140/90 mmHg, on HBP medication); hypertensive untreated, aware (BP ≥ 140/90 mmHg, not on HBP medication, aware of HBP); and hypertensive untreated, unaware (BP ≥ 140/90 mmHg, not on HBP medication, not aware of HBP)^(19)^.

Participants reported the highest level of education completed using national education categories, which were mapped to the UNESCO International Standard Classification of Education (ISCED). ISCED provides a comprehensive framework of uniform and internationally agreed definitions to facilitate comparisons of education systems across countries^(22)^. Using country-specific 2011 ISCED mappings, participant’s education was categorised into three groupings, corresponding to levels 0–2 (less than high school), 3–5 (high school) and 6–8 (college/university/postgraduate degree)^(22)^.

Salt knowledge was based on participants’ answers to the following questions: ‘Do you think that too much salt in your diet could cause a health problem?’ (yes/no), and ‘How important to you is lowering the salt in your diet?’ (very important/somewhat important/not at all important). Participants’ salt consumption behaviours were based on three questions: ‘How often do you add salt/salty sauce to your food right before eating it?’, ‘How often is salt/salty seasoning/salty sauce adding in cooking or preparing foods in your household?’ and ‘How often do you eat processed foods high in salt?’ (always, often, sometimes, rarely and never). Lastly, perceptions of salt intake were based on the following question: ‘How much salt or salty sauce do you think you consume?’ (far too much, too much, just the right amount, too little, far too little). Participants reported if they received physician’s advice to reduce salt intake: ‘During the past 3 years, has your doctor advised you to reduce salt in your diet?’ (yes/no).

Participants reported their age, sex and number of people living in household. Marital status was categorised as never married, currently married or cohabiting, and other (separated, divorced and widowed). Work status categories included employed, non-paid, homemaker, student, retired and unemployed. Participant reported their typical weekly consumption of vegetables and fruit, from which the number of daily servings was derived. Physical activity was assessed using the Global Physical Activity Questionnaire (GPAQ)^(23)^, and participants were categorised if they meet the WHO recommendation for health-enhancing physical activity of 150 min of moderate or 75 min of vigorous physical activity weekly, or equivalent^(24)^. Following BP assessment, anthropometric measurements were also taken by trained data collectors, with standing height and weight measured to the nearest 1 cm and 100 g, using a stadiometer and a calibrated balance beam scale. Overweight and obesity were based on BMI ≥ 25 and 30 kg/m^2^, respectively.

### Data analysis

To enable comparisons across countries, age was restricted to 25–65 years, resulting in the following sample sizes available for analysis: Armenia *n* 1878, Azerbaijan *n* 4700, Belarus *n* 4224, Georgia *n* 3399, Kyrgyzstan *n* 2623, Republic of Moldova *n* 3983, Tajikistan *n* 2237, Turkey *n* 4208 and Uzbekistan *n* 3203 (Table 1). To estimate the national prevalence of study outcomes (hypertension control, physician’s advice to reduce salt intake, and salt knowledge, perceptions of salt intake and salt consumption behaviours), percentages were derived using survey design weights developed by the WHO to account for multi-stage cluster design and non-response while considering the age and sex distribution of the population so that the results are nationally representative. Next, to assess differences in these percentages across education groupings (corresponding to levels 0–2, 3–5 and 6–8 of country-specific 2011 ISCED mappings), generalised linear mixed-effects regression models with the ‘logit’ link function using survey design weights and random intercepts (i.e. weighted multinomial mixed-effects regression) were applied to the data. Separate models were fitted for each outcome (hypertension control, physician’s advice to reduce salt intake, and salt knowledge, perceptions of salt intake and salt consumption behaviours), with education groupings as an independent variable, and were adjusted for age, sex, marital status, weight status (normal weight, overweight and obese), and daily servings of vegetables and fruit. The *survey* package in R 3.5.0 software (GNU General Public License) was used to account for the complex survey design, and *lme4* and *broom* packages were used for weighted multinomial mixed-effects regression models. All analyses were stratified by country to facilitate comparisons and acknowledge country-specific contexts for HBP control and cultural factors around salt intake. Significance level was set at *α* = 0·05, and statistical significance was set at *P* < 0·05.

## Results

**Fig. 1:**
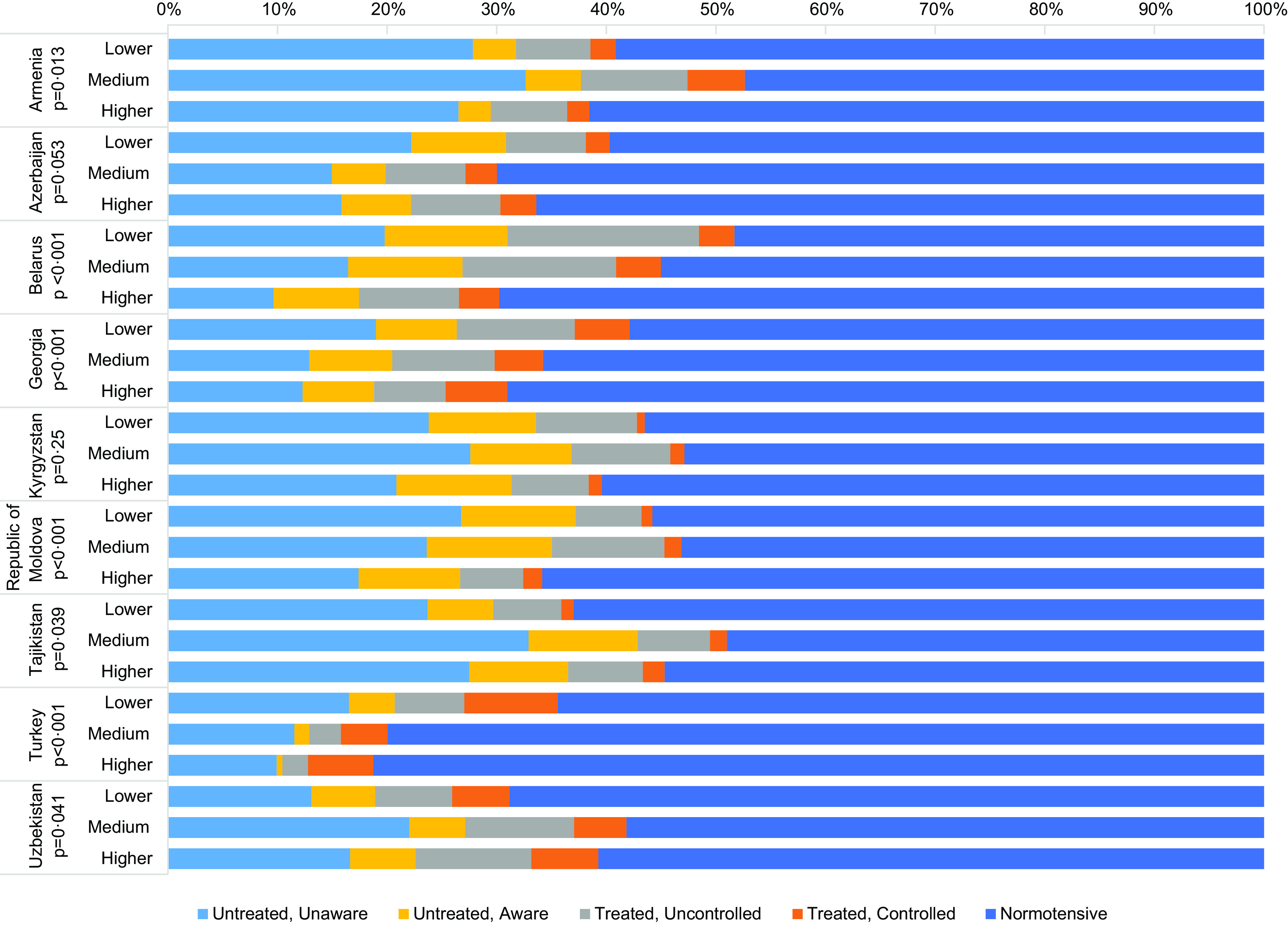
National prevalence of hypertension awareness, treatment and control according to education. Lower = less than high school. Medium = high school. Higher = college/university/postgraduate. *P-values derived from weighted multinomial mixed-effects regression models, adjusted for age, sex, marital status, weight status (normal weight, overweight and obese), and daily servings of vegetables and fruit

Participants were, on average, 41–42 years old, 45–51 % were female, and majority were married (Table 2). About one-fifth to one-quarter of participants had completed some form of post-secondary education, and this proportion varied from 13·5 % in Uzbekistan to 44·3 % in Azerbaijan. More than half were overweight or obese, with the proportion of overweight and obese participants ranging from 52·4 % in Armenia to 71·7 % in Turkey. Consumption of vegetables and fruit was well below the recommended levels, and the proportion of participants consuming at least five servings daily ranged between 12·2 % in Turkey and 39·8 % in Tajikistan. Majority of participants did not meet the WHO recommendation for weekly physical activity^(24)^, with the proportion ranging from 9·4 % in the Republic of Moldova to 41·4 % in Turkey. Over half were normotensive, with Turkey having the highest proportion of participants with normal BP (74·7 %). The remainder of the participants were hypertensive, and many were untreated and unaware of their HBP, particularly in Tajikistan, Armenia and Kyrgyzstan (30·1 %, 28·9 % and 25·6 %, respectively). HBP awareness, treatment and control varied by education (Fig. 1). In most countries, a higher proportion of participants with less than high school or high school education had worse HBP awareness, treatment and control, particularly among those with treated but uncontrolled HBP and those who were untreated and unaware of their HBP.

**Table 2:** Characteristics of study participants

	Armenia	Azerbaijan	Belarus	Georgia	Kyrgyzstan	Republic of Moldova	Tajikistan	Turkey	Uzbekistan
Age (years)									
Mean	41·5	41·8	41·9	41·9	41·8	41·8	41·8	42·1	41·7
se	0·1	0·1	0·0	0·1	0·1	0·1	0·1	0·1	0·1
Female (%)	47·4	51·4	51·1	51·5	49·7	48·4	44·8	50·5	48·8
No. people in household									
Mean	3·2	3·3	2·3	2·9	3·2	2·4	3·9	2·6	3·4
se	0·1	0·1	0·0	0·0	0·1	0·0	0·1	0·0	0·1
Marital status (%)									
Never married	11·7	10·4	14·9	14·8	5·0	7·8	2·7	11·8	5·1
Currently married or cohabiting	79·9	82·1	63·3	77·2	80·4	77·0	92·2	83·2	80·9
Other (separated, divorced and widowed)	8·4	7·5	21·7	8·0	14·5	15·2	5·1	4·9	14·0
Education (%)									
Less than high school	46·3	12·5	18·4	19·7	10·5	19·7	20·8	35·1	10·7
High school	28·2	43·2	53·4	46·5	66·5	57·9	63·4	45·9	75·8
College/university/postgraduate	25·4	44·3	28·3	33·8	23·1	22·5	15·8	19·0	13·5
Work status (%)									
Employed	44·5	49·9	80·2	43·6	48·9	58·8	36·6	45·5	46·4
Non-paid/homemaker/student	28·0	26·2	5·4	0·2	29·1	18·2	33·5	39·4	24·9
Retired	2·9	5·8	9·1	56·2	11·7	9·1	6·5	10·1	13·4
Unemployed	24·4	18·1	5·3		10·3	13·9	23·4	5·0	15·3
Weight status (%)									
Underweight	5·0	3·7	1·9	2·6	1·9	1·9	2·5	0·9	2·1
Normal weight	42·6	34·1	36·6	30·9	39·3	35·9	35·8	27·4	36·1
Overweight	30·3	39·3	36·5	33·0	34·1	35·5	39·7	39·9	33·8
Obese	22·0	22·8	25·0	33·4	24·7	26·7	22·1	31·7	28·0
Vegetables and fruit									
No. daily servings									
Mean	3·6	3·6	3·8	4·5	3·5	4·1	5·1	3·1	4·6
se	0·1	0·1	0·1	0·1	0·2	0·0	0·2	0·1	0·2
5 + servings daily (%)	25·8	24·5	26·5	38·1	26·1	35·2	39·8	12·2	32·8
Physical activity									
Does not meet WHO recommendation (%)	22·4	11·4	11·9	16·0	10·4	9·4	27·9	41·4	10·9
Average minutes per day	210·1	190·9	199·6	187·0	246·5	279·2	136·4	92·4	183·4
Physician’s advice to reduce salt intake									
Yes (%)	15·1	60·0	42·4	18·9	47·7	58·8	68·2	22·5	66·3
Hypertension awareness, treatment and control (%)									
Normotensive	56·4	67·0	57·9	65·3	54·9	56·6	52·8	74·7	59·6
Treated, controlled	3·1	2·9	3·8	4·9	1·2	1·5	1·5	6·1	5·0
Treated, uncontrolled	7·7	7·7	13·3	8·7	8·6	8·4	6·5	4·0	9·7
Untreated, aware	4·0	6·0	9·8	7·2	9·6	10·7	8·9	2·2	5·3
Untreated, unaware	28·9	16·3	15·1	13·9	25·6	22·7	30·1	13·0	20·4

The proportion of participants who received physician’s advice to reduce salt intake varied by education in Armenia, Georgia, Kyrgyzstan and Tajikistan (Table 3). A lower proportion of participants with less than high school education received physician’s advice to lower salt intake in Armenia, Georgia and Tajikistan. Importantly, there was a difference in receiving physician’s advice to lower salt intake in all countries across categories of HBP awareness, treatment and control (Table 3). Participants with treated HBP (regardless of whether it was controlled or uncontrolled) were most likely to receive physician’s advice to lower salt intake. Both normotensives and hypertensive participants who were unaware of HBP were least likely to receive advice to reduce salt intake in all countries. Among participants aware of HBP but not receiving treatment, the rates were lower than among those treated, but still higher than among normotensives and those unaware of HBP.

**Table 3:** Proportion of participants who received physician’s advice to reduce salt intake according to education and hypertension awareness, treatment and control status

	Armenia	Azerbaijan	Belarus	Georgia	Kyrgyzstan	Republic of Moldova	Tajikistan	Turkey	Uzbekistan
Education (%)									
Less than high school	12·0	51·1	38·7	14·3	46·3	56·4	61·9	24·1	67·3
High school	18·7	59·6	43·7	20·6	51·1	57·8	68·8	21·2	66·7
College/university/postgraduate	16·6	63·1	42·2	19·1	38·5	63·5	73·9	22·5	62·7
*P* ^*^	0·026	0·191	0·188	0·037	0·001	0·097	0·03	0·346	0·414
Hypertension awareness, treatment and control (%)									
Normotensive	10·8	53·3	37·5	13·9	43·2	56·8	66·7	18·7	64·8
Treated, controlled	43·5	79·2	59·6	32·2	69·1	73·9	90·9	43·9	71·3
Treated, uncontrolled	35·7	73·8	60·8	43·3	73·6	76·3	79·6	58·9	76·4
Untreated, aware	25·5	68·5	55·1	30·1	61·6	61·6	64·4	40·7	71·0
Untreated, unaware	12·9	57·2	31·9	19·2	42·1	53·9	67·9	19·3	63·6
*P* ^*^	<0·001	<0·001	<0·001	<0·001	<0·001	<0·001	0·001	<0·001	0·031

* From weighted multinomial mixed-effects regression models, adjusted for age, sex, marital status, weight status (normal weight, overweight and obese), and daily servings of vegetables and fruit.

**Fig. 2:**
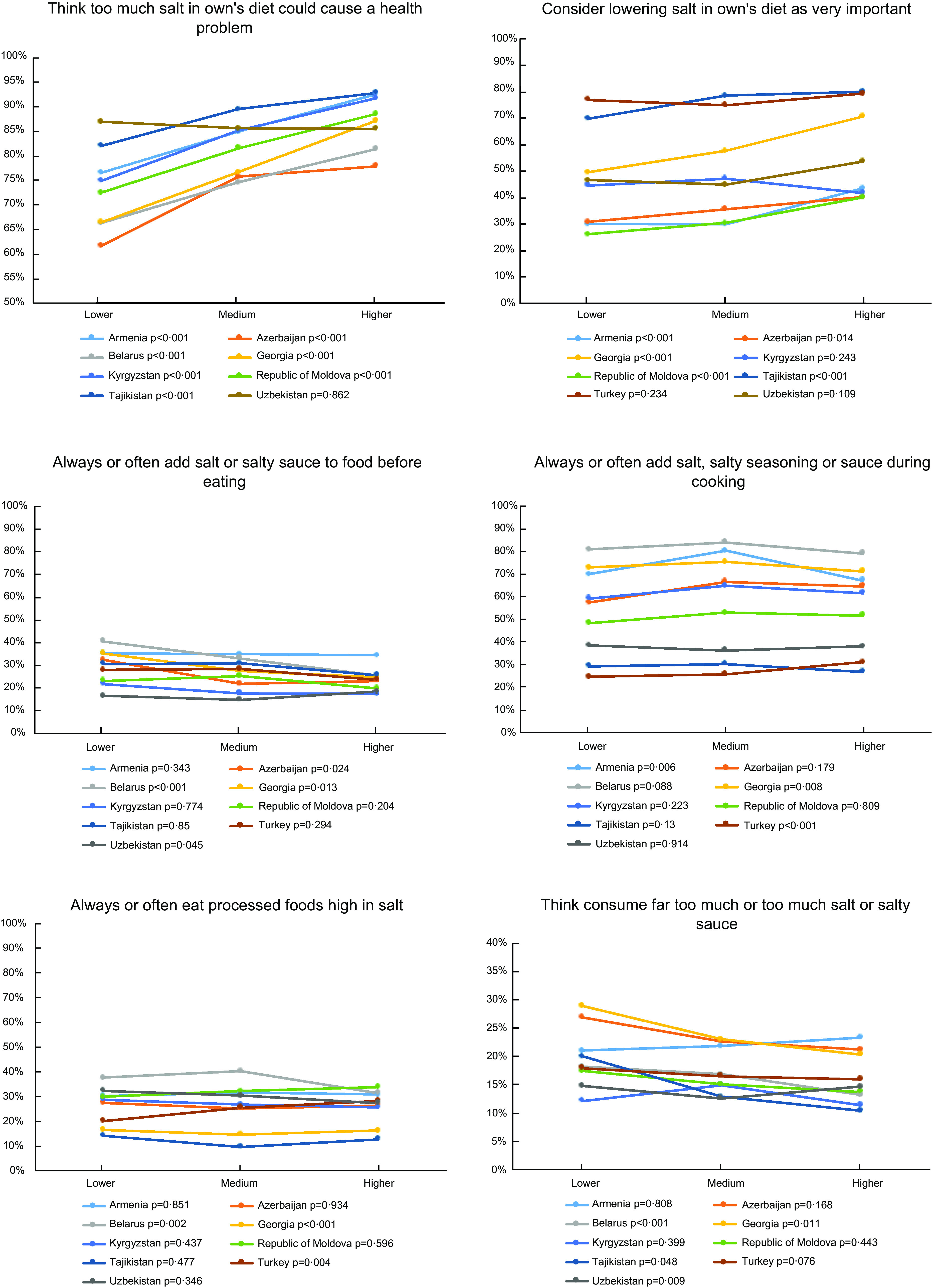
National prevalence of salt knowledge, perceptions of salt intake and salt consumption behaviours according to education. Lower = less than high school. Medium = high school. Higher = college/university/postgraduate. *P-values derived from weighted multinomial mixed-effects regression models, adjusted for age, sex, marital status, weight status (normal weight, overweight and obese), and daily servings of vegetables and fruit

Participants with lower education frequently reported lower levels of salt knowledge (Fig. 2). Salt knowledge (‘Think too much salt in own’s diet could cause a health problem’ and ‘Consider lowering salt in own’s diet as very important’) improved consistently with higher education in almost all countries. In terms of perceptions of salt intake, a higher proportion of participants with lower education considered that they consume far too much or too much salt in Belarus, Georgia, Tajikistan and Uzbekistan. However, salt consumption behaviours did not show a consistent education gradient. Participants with lower education reported more frequently that they always/often add salt before eating than those with higher education in Azerbaijan, Belarus and Georgia. Always/often adding salt during cooking was more prevalent among participants with higher than lower education in Turkey (as well as frequent consumption of processed foods high in salt), and among participants with medium education in Armenia and Georgia.

**Fig. 3:**
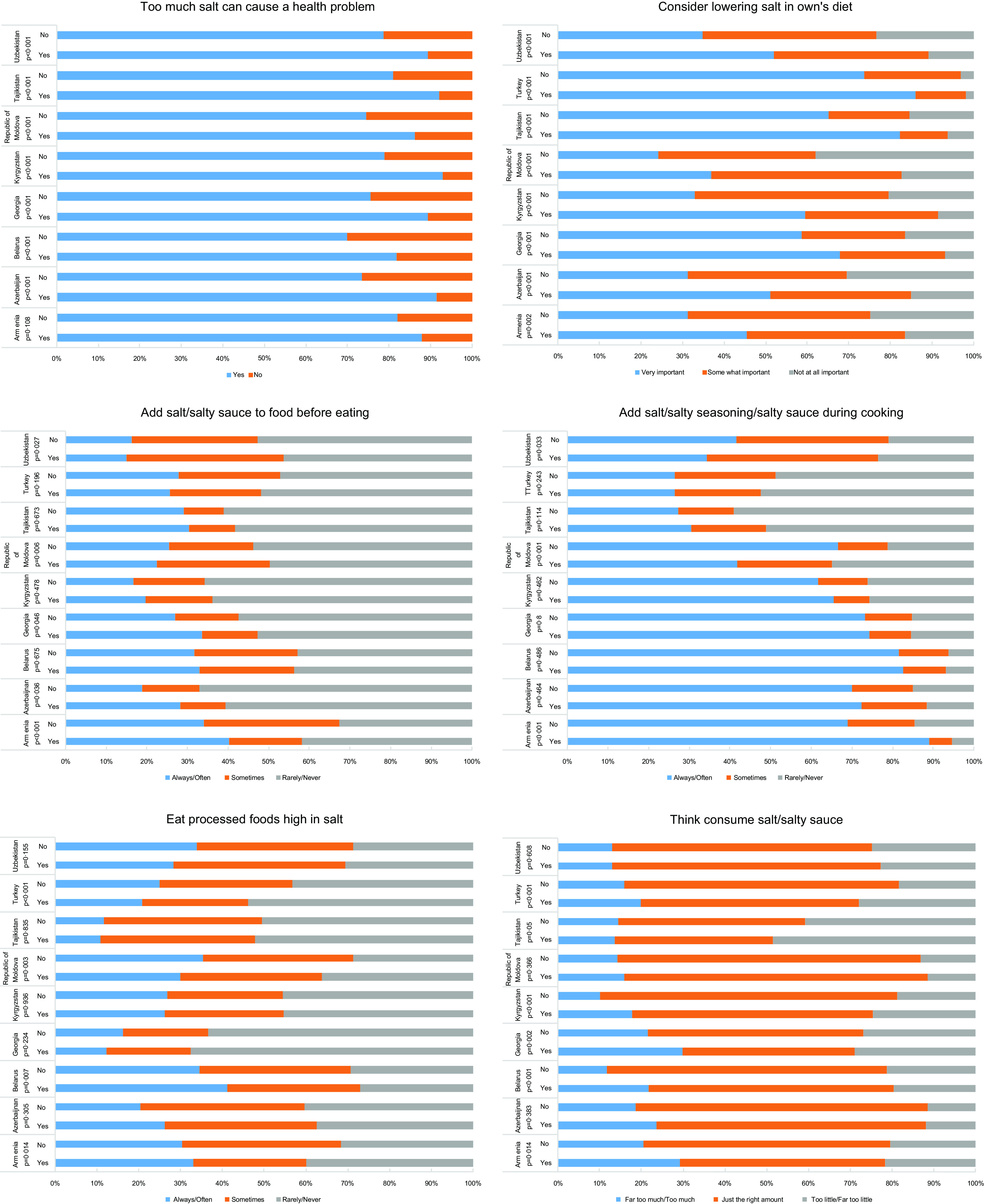
National prevalence of salt knowledge, perceptions of salt intake and salt consumption behaviours according to physician’s advice to reduce salt intake. Yes = received physician’s advice to reduce salt intake during the past 3 years. No = did not receive physician’s advice to reduce salt intake during the past 3 years. *P-values derived from weighted multinomial mixed-effects regression models, adjusted for age, sex, marital status, weight status (normal weight, overweight and obese), and daily servings of vegetables and fruit

Salt knowledge was higher among those receiving physician’s advice to reduce salt intake (Fig. 3). In all countries, a higher proportion of participants reported that too much salt in their own diet could cause a health problem, and that lowering salt in their own diet is very important if they received physician’s advice to lower salt intake. In terms of perceptions of salt intake, a much higher proportion of participants reported thinking they consume far too much or too much salt if they received physician’s advice to reduce salt intake in Armenia, Belarus, Georgia, Kyrgyzstan and Turkey. Nonetheless, frequent salt consumption (always/often adding salt to food before eating or during cooking and eating processed foods high in salt) did not show a consistent improvement among those receiving physician’s advice to reduce salt intake. Better salt consumption behaviours among participants who received physician’s advice to reduce salt intake were observed only in the Republic of Moldova, Turkey and Uzbekistan.

## Discussion

This study reports for the first time on nationally representative educational inequalities in HBP awareness, treatment and control as well as in salt knowledge, perceptions and behaviours from nine countries in Eastern Europe and Central Asia. Four key findings emerge from this study. First, the national prevalence of hypertension was high, and HBP treatment and control rates varied by country but were very low in general. Substantial differences exist in HBP awareness, treatment and control across the educational groupings, with participants with lower levels of education having lower levels of HBP awareness, treatment and control. Second, participants with diagnosed and treated HBP received the advice by a health care professional to reduce salt intake more often, although the coverage of physician’s advice on salt reduction was low in all groups. Third, knowledge of harmful effects of salt on health was lower among participants with lower education, but the education gradient in salt consumption behaviours was less pronounced and the direction of association was inconsistent. Lastly, knowledge of harmful effects of salt was more prevalent among participants who received advice on salt reduction from health care professionals, but the impact of physician’s advice was not evident for salt consumption behaviours.

The suboptimal HBP awareness, treatment and control, whereby many participants with diagnosed hypertension do not have their HBP controlled despite being treated, and a large proportion of hypertensive participants either do not have their HBP treated or are unaware of their HBP, is concerning but corroborates the emerging evidence from low- and middle-income settings^(25,26)^. While evidence on HBP awareness, treatment and control in low- and middle-income settings is scant, the findings align with the rates observed in high-income countries in the 1980s and early 1990s, whereby treatment rates were at most 40 % and control rates were less than 25 %^(5)^. Importantly, our study demonstrated a large and consistent educational gradient in HBP awareness, treatment and control^(26,27)^. Overall, results highlight that improvements in HBP awareness, treatment and control are urgently needed in low- and middle-income settings^(28)^. Indeed, evidence from high-income countries suggests that hypertension awareness, treatment rate and control rate can be improved rapidly following implementation of national programmes for hypertension education and/or health check-ups and identifies tailored knowledge dissemination (targeting socio-economically disadvantaged, hard-to-reach population subgroups) as an important ingredient of success of national education programmes^(5)^.

Results from this study identify important gaps in the coverage of advice on salt reduction by health care professionals. Physician’s advice is a well-established strategy for improving HBP control^(29,30)^. However, we found that the coverage of physician’s advice to reduce salt intake was less frequent among participants with lower education as well as those who were hypertensive but were untreated or unaware of their HBP. This lack of physician’s advice poses a double threat, since a strong socio-economic gradient (whether based on education and other dimensions of socio-economic status (SES) such as income or occupation) exists in the prevalence of HBP, and lower SES groups bear disproportionately higher rates of CVD mortality, compared with higher SES groups^(18,31)^. The findings from this study corroborate this evidence and highlight the need for improvements in the population-wide coverage of medical advice and awareness raising on salt reduction, with greater emphasis on targeting those with lower education and untreated HBP (e.g. through primary health care multidisciplinary collaboration between physicians, nurses and pharmacists^(32,33)^ and pharmacy-based interventions^(34)^). The feasibility of adopting evidence-based approaches to implement in countries in Eastern Europe and Central Asia should be investigated using modern implementation science methodologies^(35,36)^.

In this study, we documented an education gradient in salt knowledge, which may contribute to the high burden of CVD among lower SES groups. Overall, the personal risk associated with salt consumption was not salient for many participants, and the perception of personal risk was socio-economically patterned. Risk reduction knowledge is often strongly socio-economically patterned and is lowest among those with low SES^(37)^. Recent evidence from Italy and the UK in general populations also find socio-economic differences in salt knowledge and awareness of salt reduction guidelines^(38,39)^. Since a lack of knowledge is commonly identified as a barrier to salt restriction in adults with hypertension^(40)^, and better salt awareness and self-control of salt intake have been linked to lower prevalence of hypertension in general adult populations^(41)^, our findings make an important contribution to the nascent literature on socio-economic inequalities in salt knowledge in Eastern European and Central Asian countries.

Moreover, the translation of improvements in knowledge and perceptions into better lifestyle behaviours depends on individual social and economic resources which may or may not allow individuals to benefit from such interventions^(42)^. Our findings from nine countries in Eastern Europe and Central Asia reveal the lack of consistent patterns in salt consumption behaviours across education groupings, despite an education gradient in knowledge and perceptions of one’s own salt intake. Nonetheless, previous studies have demonstrated a socio-economic gradient in salt consumption in the UK, Italy and Montenegro when salt intake was measured through dietary recall or a 24-h urine collection^(43–45)^. Overall, the study findings suggest that, in addition to evidence-informed programmes to improve salt knowledge^(13,46)^, other approaches are needed to optimise the effectiveness of country-specific salt reduction strategies and maximise population impact on behaviour change^(47)^. Specifically, legislative and regulatory interventions that engage industry to limit salt content in processed foods through product reformulation are also needed to facilitate development and implementation of comprehensive population-wide salt reductions strategies^(48)^ and to reduce health inequalities^(49)^.

The strengths of this study include large and nationally representative samples, standardised and state-of-the-art questionnaire as well as objective and standardised measurement of biological risk factors including BP, weight and height. Several limitations warrant consideration. Although the data were adjusted for non-response rate, there is a possibility that non-respondents have different characteristics than respondents in the surveys. Better knowledge of salt effects on health lead to better health outcomes via diets low in salt. Measures of salt knowledge, participants’ perceptions of salt intake and salt consumption behaviours were all self-reported. We cannot rule out the possibility of recall or social desirability bias introducing measurement error. The WHO STEPS surveys do not have a reliable objective measure of salt/Na intake at the individual level. It is possible that an education gradient in salt consumption may be detected if salt intake was measured through dietary recall or a 24-h urine collection. We are also not able to disentangle whether the gaps in the coverage of physician’s advice to reduce salt intake among participants with lower education as well as those who were hypertensive but were untreated or unaware of their HBP may be due to issues of reduced frequency of accessing health care or health promotion services, recall bias or physician’s bias in providing care. Future research should investigate the sources of the discrepancies in the coverage of physician’s advice on salt reduction by health care professionals. Finally, although education is a robust indicator of SES (defined as one’s material and social standing relative to others), only inequalities based on education, but not other dimensions of SES (e.g. income and occupation), could be considered due to the data parameters.

The findings suggest that HBP awareness, treatment and control were low in Eastern European and Central Asian countries, and there was a strong education gradient in HBP awareness, treatment and control as well as salt knowledge and perceived intake. Results from this study underscore the urgent need for enhancements in public and patient knowledge and awareness of HBP and its risk factors, targeting socio-economically disadvantaged groups with lower education to help alleviate the high and growing HBP burden in low- and middle-income countries in the WHO European Region and worldwide. The findings reveal educational inequalities in physician’s advice on salt reduction. Given that physician’s advice is a promising intervention strategy, future implementation science research studies should investigate its potential to optimise the delivery of existing brief interventions and patient education programmes in the primary health care settings by reaching the most high-risk and socio-economically disadvantaged segments of the population while considering local primary health care systems, level of development, cultural factors and resources available for health promotion. Other potential avenues for reducing salt intake at the population level may include legislative and regulatory approaches, which rely less on individual uptake and have been shown to contribute to health improvements in an equitable manner^(50)^.
